# Upregulation of Oxytocin Receptor in the Hyperplastic Prostate

**DOI:** 10.3389/fendo.2018.00403

**Published:** 2018-08-03

**Authors:** Zhuo Li, He Xiao, Kebing Wang, Yuelan Zheng, Ping Chen, Xinghuan Wang, Michael E. DiSanto, Xinhua Zhang

**Affiliations:** ^1^Zhongnan Hospital of Wuhan University, Wuhan, China; ^2^Shenzhen Key Laboratory for Endogenous Infection, Department of Urology, Shenzhen Sixth People's Hospital, The Sixth Affiliated Hospital of Shenzhen University Health Science Center, Affiliated Shenzhen Sixth Hospital of Guangdong Medical University, Shenzhen, China; ^3^Department of General Surgery, Children's Hospital of Shenzhen, Shenzhen, China; ^4^Departments of Biomedical Sciences, Surgery of Cooper Medical School of Rowan University, Camden, NJ, United States

**Keywords:** oxytocin, receptor, prostate, benign prostatic hyperplasia, androgen, estrogen

## Abstract

**Background:** The etiology of benign prostatic hyperplasia (BPH) is complex, both age and androgen are thought to be important. However, the failure of androgen blockade treatments suggests other paracrine/autocrine factors involved in BPH. Oxytocin was found to have a paracrine/autocrine role in prostate in recent years. The influence of BPH on prostatic oxytocin receptor (OTR) expression has never been studied.

**Material and methods:** A testosterone-estradiol induced rat model of BPH was employed and human hyperplastic prostate specimens were harvested. Expressions of OTR, α_1_-adrenoreceptor subtypes and nitric oxide synthase isoforms were determined via real-time RT-PCR. OTR was further analyzed with Western-Blotting and histological examination. Subsequently, rat epithelial cells, human stromal cells and epithelial cells were cultured *in vitro* and treated with gradient concentrations of OT from 1 to 5 days. Cell proliferation was tested by Cell Counting Kit-8 and Flow Cytometry.

**Results:** The rat BPH model was validated with significant increased prostate weight. H-E stain revealed a different histopathology between human and rat BPH. Masson's trichrome staining demonstrated that smooth muscle (SM) cells, epithelium cells and collagen fibers were simultaneously augmented in this rat BPH model and human BPH samples. OTR mainly localized in epithelium in rat prostate whereas it mainly localized in stroma in human prostate. OTR gene was upregulated 3.3-fold in rat BPH and 3.0-fold in human BPH, along with increased expression of 2.0-fold α_1a_ARs and 3.0-fold eNOS for rat BPH and 5.0-fold α_1a_ARs for human BPH. The expression of OTR protein was upregulated 1.4-fold in rat BPH and 3.9-fold in human BPH, respectively. Increased concentrations of exogenous OT can accelerate proliferation of rat epithelial cells and human stromal cells but has no impact on human epithelial cells *in vitro*. Flow Cytometry showed oxytocin could significantly increase G_2_/M period cell number.

**Conclusions:** Our novel data demonstrates a significant and previously undocumented upregulation of OTR in both rat and human BPH. Moreover, exogenous OT accelerates proliferation of rat prostate epithelial cells and human prostate stromal cells. It is suggested OTR is involved in the development of BPH and OT regulatory system could be a potential new target for the BPH treatment.

## Introduction

Benign prostatic hyperplasia (BPH) is one of the most reported clinical complaints in adult men and can lead to lower urinary tract symptoms (LUTS) (1). Longitudinal data suggests an annual prostate growth rate of 1.6% as measured by transurethral ultrasonography (2). The prevalence of BPH is approximately 50% for men in their fifties and reaches to 80% for men in their eighties (3). Despite its high prevalence, the pathogenesis of BPH is still unclear. Androgens were once thought of as important factors for BPH, especially dihydrotestosterone (DHT) (4), but failure of anti-androgen treatments to completely halt the progress of prostatic hypertrophy suggests other factors may also be involved in the regulation of prostatic growth. In the 1990s, Suzuki et al. found testicular function decreased in aging men, leading to a decline of testosterone (T) levels in plasma, while the conversion of androgen to estrogen increased, resulting in an unchanged estrogen level and an augmented proportion of estrogen to androgen (5). In a quantitative study, the ratio of estrogen/androgen is 1:150 in middle-aged men whereas it rises to 1:80–120 in aging men (6). Furthermore, the proportion is 1:8 in hyperplastic prostate tissue, suggesting estrogen also plays a role in the development of BPH (6).

It has been documented that estrogen can modulate the peptide oxytocin (OT) system, a classical female neurohypophysis hormone playing a role in parturition and milk ejection. Interestingly, OT also exists in the male reproductive tract, which was first found both in human and rat testis by Nicholson et al. (7). Early studies have confirmed OT has two effects on male erectile function, it promotes erectile function in the central nervous system (8) whereas it inhibits erectile function in the corpus cavernosum (9). Within the prostate, the OT concentration is far higher than in plasma and OT mRNA has been detected, suggesting OT may be synthesized and function locally (10). The essential role of OT is vasoconstriction, such as uterine contraction and milk ejection for females, orgasm and detumescence post-ejaculation for males. Increased tone and volume of the prostate are the two major pathophysiological mechanisms for BPH. An elevated estrogen/androgen ratio would upregulate the OT system and related contractility in the prostate. On the other hand, mitogenic effects of OT have been observed in the rat prostate. The volume of the prostate, the epithelial cell volume and the diameter of the acinar lumens were all increased significantly following daily administration of OT for 10 days in castrated rats compared with untreated castrated rats (11). In a recent study, the OT level was found highly increased in the prostate tissue from BPH patients and induced prostatic cell proliferation (12). The mechanism of these growth effects remains unknown. The peptide may have a direct mitogenic effect, since OT has been demonstrated to stimulate growth in osteoblasts and human small cell carcinoma of lung (13), as well as in liver and adrenal cells *in vitro* (14).

OT exerts its role through combining and activating the OT receptor (OTR), which is a seven transmembrane-domain poly-peptide belonging to the rhodopsin-type class 1 G-protein coupled receptor (GPCR) family (15). The evidence of OTR localized within the prostate was first reported by Einspanier et al., in the marmoset prostate (16). Subsequently, it was found existing in human prostate (17). However, various distributions were reported in the prostate (18). Moreover, the differences of OTR mRNA and protein expression in normal prostate and hyperplastic tissue have not yet been determined. In the current study, we used a rat BPH model and human hyperplastic prostate tissue to investigate the expression of OTR gene and protein, as well as genes involved in the major pathways regulating smooth muscle (SM) tone. We further cultured prostatic epithelial cell and stromal cell *in vitro* and treated them with increasing concentrations of OT.

## Materials and methods

### Animals and tissues

A total of 30 specific-pathogen-free (SPF) grade male Wistar rats (12 weeks) weighing 225–275 g were used. Rats were randomly divided into two groups. One group (*n* = 15) was treated with 0.1 ml sesame oil as controls and the other (*n* = 15) were subcutaneously injected with 2 mg/d testosterone propionate (19) (Tianjin Jinyao Amino Acid Co., Ltd, Tianjin, China) mixed with estradiol benzoate (Tianjin Jinyao Amino Acid Co.) at the ratio 100:1 for 28 days. The daily dose of testosterone propionate and estradiol benzoate is 2 mg and 0.02 mg, respectively. The daily volume injected is 0.1 ml. Rats were weighed and sacrificed under anesthesia on day 29. All ventral prostatic lobes, seminal vesicles and bladder were harvested and weighed. Nine samples from young brain-dead men (mean age, 29.1 ± 1.7 years old) undergoing organ donation were obtained as controls and nine BPH case samples were obtained from patients (mean age, 67.7 ± 2.1 years old) undergoing cystoprostatectomy for infiltrating bladder cancer without prostate infiltration. All human samples were obtained after the approval of the Hospital Committee for Investigation in Humans and after receiving written informed consent from all patients or their relatives when needed. Prostate tissues were divided into three strips and were respectively stored in RNA Sample Protector (Takara Bio. Inc., Otsu, Shiga, Japan) for PCR analysis, 10% neutral buffered formalin for histological examination, liquid nitrogen for Western-Blotting analysis. All animal protocols were approved by the Animal Experiment Center of Zhongnan Hospital of Wuhan University and the human study was conducted in accordance with the principles of the Declaration of Helsinki.

### Human prostatic cell lines and rat primary epithelial cell

SV40 large-T antigen-immortalized stromal cell line WPMY-1 (Cat. #GNHu36) was purchased from the Stem Cell Bank, Chinese Academy of Sciences in Shanghai, China. Human benign prostatic enlargement epithelia cell line BPH-1 (Cat. #BNCC339850) was purchased from the Procell Co., Ltd. in Wuhan, China. Identification of the cell lines was performed at the China Center for Type Culture Collection in Wuhan, China. Rat primary epithelial cell was purchased from Wuhan Aisenyuan Technology Co., Ltd., Wuhan, China. Identification of rat epithelial cell was performed at Wuhan Aisenyuan Technology Co., Ltd., Wuhan, China. The BPH-1 cells were cultured in RPMI-1640 medium (Gibco, China) containing 10% fetal bovine serum (FBS) (Gibco, Australia), WPMY-1 cells were cultured in DMEM medium (Gibco, China) containing 1% penicillin G sodium/streptomycin sulfate and 5% FBS in a humidified atmosphere consisting of 95% air and 5% CO_2_ at 37°C. Rat primary epithelial cell was cultured in medium 199 (Gibco, China) containing 10% fetal bovine serum (FBS) (Gibco, Sydney, Australia) in a humidified atmosphere with 5% CO_2_ at 37°C. After culture for 24 h, rat epithelial cells, BPH-1 cells and WPMY-1 cells were treated with decreasing gradient concentrations of OT from 10^−6^ U/ml to 10^−12^ U/ml for 1, 2, 3, 4, and 5 days, respectively. Cells were repeatedly cultured six times at each concentration and duration. CCK-8 (Shanghai Dojindo Chemical Technology Co., Ltd., Shanghai, China) was used for cell number measurement. Optical Density (OD) was examined at 450 nm by a microplate reader (Thermo Fisher Scientific Inc., MA, USA).

### Cell immunofluorescence and flow cytometry analysis

For cell immunofluorescence microscopy, cells were cultured as aforementioned, followed by seeding on 12 mm coverslips and washing by ice cold phosphate-buffered saline (PBS, pH = 7.4). The coverslips were then fixed with 4% paraformaldehyde (PFA) for 30 min, followed by treated in 0.1% Triton X-100 and blocked in goat serum for 30 min at room temperature. OTR primary antibody (Abcam, Cambridge, UK; ab181077) and SM Myosin primary antibody (Abcam, ab683) were used for incubation. The secondary antibody employed to visualize the localization of OTR and Myosin was Cy3-conjugated goat anti-rabbit IgG (Technology, Cat. #4413) and FITC-conjugated goat anti-mouse IgG (Technology, Cat. #4407), respectively. DAPI was used for staining the nucleus. Visualization was done with a Laser Scanning Confocal Microscope (Olympus, Tokyo, Japan). For cell cycle analysis, rat primary epithelial cells and WPMY-1 cells were cultured with 0 U/ml and 10^−8^ U/ml OT for 48 h, respectively. 1 × 10^6^ cells were harvested and fixed in 70% ice cold ethanol at −20°C for overnight. After centrifugation, pellets were resuspended with PBS containing 50 μg/ml propidium iodide and 0.1 mg/ml RNase A in the dark. After incubation at 37°C for 30 min, the DNA content distribution was analyzed by Flow Cytometry (Beckman, Cat. #FC500).

### H&E staining and masson's trichrome staining

Rat and human prostate tissues fixed in 10% neutral buffered formalin for 24–36 h were processed routinely for paraffin embedding. The paraffin-embedded tissue sections (4 μm) were stained with hematoxylin and eosin using standard techniques. The paraffin sections were deparaffinized in xylene, followed by graded alcohols. Masson composite staining solution (Fuzhou Maxim Biotech Co., Ltd., Fuzhou, China) was added dropwise for 10 min. The sections were subsequently washed with distilled water, differentiated in phosphomolybdic-phosphotungstic acid solution for 10 min, and incubated with blue staining solution for 5–10 min. Next, the sections were rinsed briefly in distilled water and differentiated in 1% acetic solution for 2 min. After being dehydrated quickly through 95% alcohol, absolute alcohol, the sections were cemented using neutral gum for observation. Using this procedure, prostatic stroma SM cells were stained red, collagen fibers were stained blue and epithelial cells were stained orange. In each sample, we analyzed three areas under magnification (× 100). The choice of three fields was randomized without specific areas of a demarcated slide. The area percentage of SM, collagen fibers and glandular epithelium were quantitated with Image Pro Plus 5.0, respectively.

### Immunohistochemistry and immunofluorescence

For immunohistochemistry, sections were deparaffinized in xylene, followed by graded alcohols. Antigen retrieval was performed in 10 mM sodium citrate buffer (pH 6.0) and heated to boil. Sections were kept in boiled buffer for 2 min. Endogenous peroxidase activity was blocked by using 3% H_2_O_2_ solution at room temperature for 10 min. Then sections were incubated with 15% normal goat serum for 15 min at room temperature to block non-specific binding. OTR primary antibody (1:500) (Abcam, ab181077) was applied to the sections on the slides and incubated in a humidified chamber at 4°C overnight. Then the sections were stained by routine immunohistochemistry methods. Negative controls were performed for all samples by omitting the primary antibody. Rat postpartum uterus tissue was used as a positive control for OTR staining. All the stained sections were imaged using an Olympus-DP72 light microscope (Olympus). For immunofluorescence, tissues were sectioned in 10 μm thick slices and thawed, mounted onto glass slides using a cryostat (Leica CM 1850, Wetzlar, Germany), air-dried, and fixed for 10 min in ice cold acetone. Slides were washed in PBS and incubated for 2 h in a mixture of PBS supplemented with 0.2% Triton X-100 and 0.1% bovine serum albumin, followed by incubation overnight with the primary antibody (1:100). The secondary antibody employed to visualize the localization of OTR was Cy3-conjugated goat anti-rabbit IgG (1:1,000). DAPI was used for staining the nucleus. Negative controls were performed for all samples by omitting the primary antibody. Visualization was done with a Laser Scanning Confocal Microscope (Olympus).

### Total RNA extraction and real-time RT-PCR

Total RNA was isolated from the frozen tissues using Takara RNAiso Plus (Takara Bio. Inc.) according to the manufacturer's protocol. Genomic DNA (gDNA) was removed and cDNA was reverse-transcribed by using Takara PrimeScript^TM^ RT reagent Kit with gDNA Eraser (Takara Bio. Inc.) in a T100^TM^ Thermal Cycler System (BioRad, USA). The experimental protocol utilized was first gDNA removal (42°C 2 min), followed by reverse transcription (37°C 15 min, 85°C 5 s). Subsequently, all samples were amplified by a 25 μl reaction volume in a CFX96^TM^ Real-time PCR Detection System (BioRad), using SYBR® *Premix Ex* Taq^TM^ II (Takara Bio. Inc.). All samples were run in triplicate. OTR, α_1_-adrenoreceptor subtypes (α_1a_ARs, α_1b_ARs, α_1d_ARs), endothelial NOS (eNOS) and neuronal (nNOS) were investigated. The amplification program was repeated for 40 cycles (OTR: 95°C 10 s, 58°C 30 s, 72°C 30 s. α_1a_ARs, α_1b_ARs, α_1d_ARs, eNOS, and nNOS: 95°C 5 s, 60°C 50 s). Primer sequences are shown in Table 1. For relative quantification, gene expression was normalized to expression of the glyceraldehyde 3-phosphate dehydrogenase (GAPDH housekeeping gene) and compared by 2^−ΔΔ*CT*^ method.

**Table 1:** Primer sequences used to amplify target genes by real-time RT-PCR.

**Target gene**	**Primer sequence**	**Target gene**	**Primer sequence**
Rat		Human	
OTR		OTR	
Forward	5′-TGCTCTGCTCGTTACCTGAA-3′	Forward	5′-AATTGGGTCAGGAAGTCCAGT-3′
Reverse	5′-CATGCTGAAGATGGCTGAGA-3′	Reverse	5′-CACTTTGAGGTCAGGAGGATCT-3′
α_1a_AR		α_1a_AR	
Forward	5′-TCTGCATCATCTCCATCGAC-3′	Forward	5′-CCACTTCAACGAAAACCACCA-3′
Reverse	5′-GACCAAAGAAAGCACCCAGA-3′	Reverse	5′-AAAATCCCCTCACTTCCATCAA-3′
α_1b_AR		α_1b_AR	
Forward	5′-CCAGGAGTTCCATAGCTGTCAAAC-3′	Forward	5′-TGGGGAGAGTTGAAAAATGC-3′
Reverse	5′-CCGACTACAATGCCCAAGGT-3′	Reverse	5′-GATGGCAAAGAGGATGAAGG-3′
α_1d_AR		α_1d_AR	
Forward	5′-TGCGCCACTCGCTCAA-3′	Forward	5′-GTCTTCGTGCTCTGCTGGTT-3′
Reverse	5′-CCAAAGCAGAGCCAGAATGG-3′	Reverse	5′-GCTGGAACAGGGGTAGATGA-3′
eNOS		eNOS	
Forward	5′-CACCCACTGAGCAGTATTGG-3′	Forward	5′-GGCATCACCAGGAAGAAGAC-3′
Reverse	5′-CCTGGGAACCACTCCTTTTG-3′	Reverse	5′-TCGGAGCCATACAGGATTGT-3′
nNOS		nNOS	
Forward	5′-GGCAAACATGACTTCCGAGTGT-3′	Forward	5′-CCCTGAGAATGGGGAGAAAT-3′
Reverse	5′-CCCCAAGGTAGAGCCATCTG-3′	Reverse	5′-GCTGTTGAATCGGACCTTGT-3′
GAPDH		GAPDH	
Forward	5′-ACAGCAACAGGGTGGTGGAC-3′	Forward	5′-AGAAGGCTGGGGCTCATTTG-3′
Reverse	5′-TTTGAGGGTGCAGCGAACTT-3′	Reverse	5′-AGGGGCCATCCACAGTCTTC-3′

### SDS-PAGE and western-blotting analysis

Proteins were extracted from frozen samples using the Radio Immunoprecipitation Assay Lysis Buffer (Shanghai Beyotime Bio. Co., Shanghai, China). One hundred μg of each sample was eletrophoresed on a 10% sodium dodecyl sulfate-polyacrylamide (SDS-PAGE) gel (Wuhan Boster Biological Technology Ltd., Wuhan, China) and transferred to polyvinylidene fluoride (PVDF) menbrane (Millipore, Billerica, MA, USA) using a BioRad wet transfer system. The membrane was blocked for 2 h at room temperature with Tris-buffered saline with 0.1% Tween (TBST) containing 5% non-fat dry milk solution. The membrane was incubated overnight with primary OTR antibody (Rabbit monoclonal to OTR, ab181077) at dilution of 1:1,000 (Abcam). Membranes were washed with TBST three times and incubated at room temperature for 1 h with an IRDye 800CW-conjugated goat anti-rabbit IgG (LI-COR, Lincoln, USA) at dilution of 1:15,000. After washing, the blots were visualized by using Li-Cor Odyssey Imager (Li-Cor). The bands were quantified by densitometry using Li-Cor Odyssey software. A monoclonal rabbit antibody against GAPDH (1:10,000; Abcam, ab181602) was used as a control to ascertain equivalent loading. All samples analyses were independently repeated for 3 times.

### Statistical analysis

Results are expressed as mean ± standard deviation (SD) in table and mean ± standard error of mean (SEM) in bar graph. Student's *t*-test was performed by Excel software (two sample treatments compared) to analyze the differences between two groups. *P* < 0.05 was considered statistically significant.

## Results

**Figure 1:**
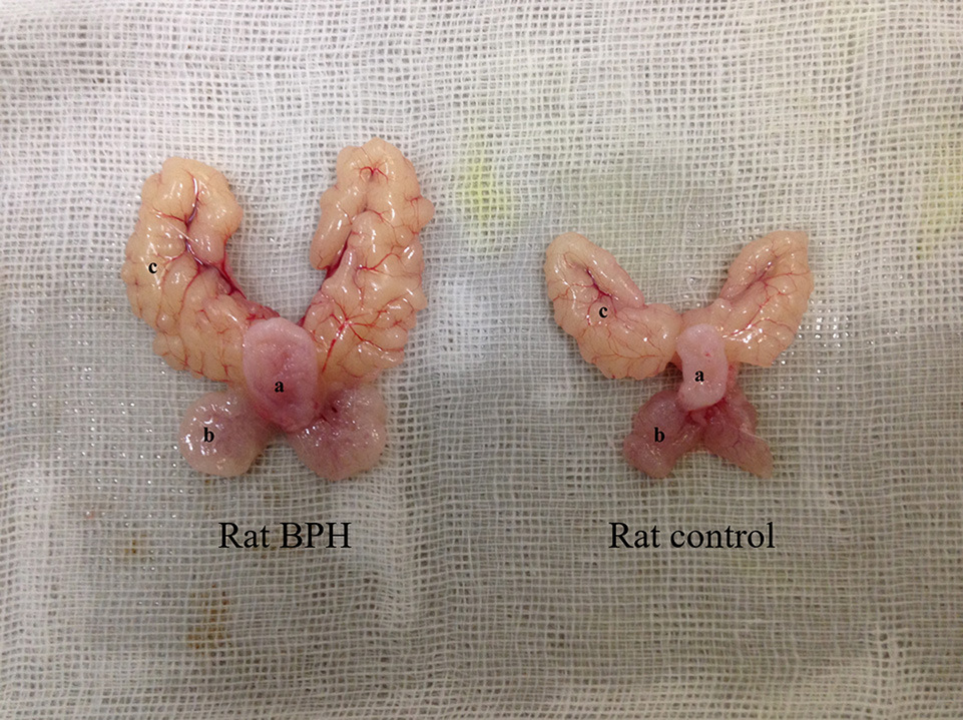
Typical photograph from a BPH and control rat. **(a)** bladder, **(b)** ventral prostate, **(c)** seminal vesicle.

The testosterone (T)-estradiol (E)-supplementation rat model of BPH was validated through increased weight of the ventral prostate and seminal vesicles (Figure 1 and Table 2) by 1.9-fold and 2.2-fold (*P* < 0.01), respectively. Accordingly, the prostate index [prostate wet weight (mg)/body weight (g)] was calculated with a 2.1-fold increase observed in the BPH group (Table 2, *P* < 0.01). The body weight of BPH rats was significantly decreased (Table 2, *P* < 0.01), which may be ascribed to the physiological effect of T.

**Table 2:** Variation of biometric and physiological parameters in control and BPH rats.

**Group**	**Body weight(g)**		**Ventral prostate weight(mg)**	**Seminal vesicles weight(mg)**	**Prostate index**
	**Initial**	**Final**			
Control	246.4 (14.8)	361.1 (20.4)	548.2 (91.7)	1023.5 (180.7)	1.5 (0.2)
BPH	249.1 (17.3)	336.7 (22.3)^**^	1030.4 (141.2)^**^	2256.3 (259.5)^**^	3.1 (0.5)^**^
*P*-value	0.656	0.005	0.000	0.000	0.000

*P-values calculated by unpaired t test. Data are mean ± SD*.

** *P < 0.01 vs. control*.

**Figure 2:**
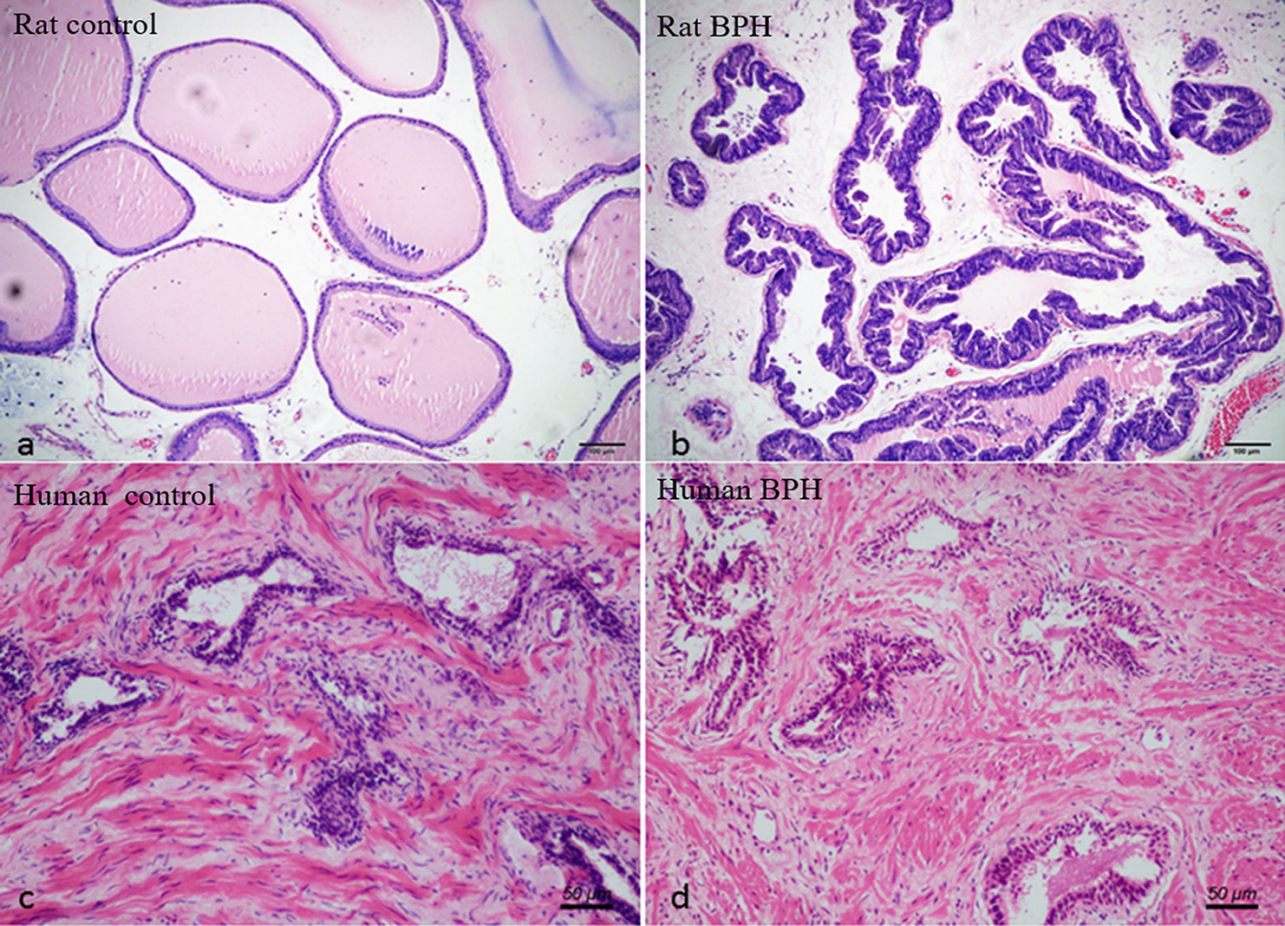
Histological examination of prostate. H-E staining of prostate tissue. **(a)** Normal rat prostate. **(b)** BPH rat prostate. Hyperplastic prostate occurred mainly in the epithelial compartment and typical features of glandular hypertrophy were observed including papillary fronds protruded into the glandular cavities and the epithelial layer thickened. (magnification × 100). **(c)** Normal human prostate. **(d)** BPH human prostate. An obvious stromal hyperplasia was observed (magnification × 200).

**Figure 3:**
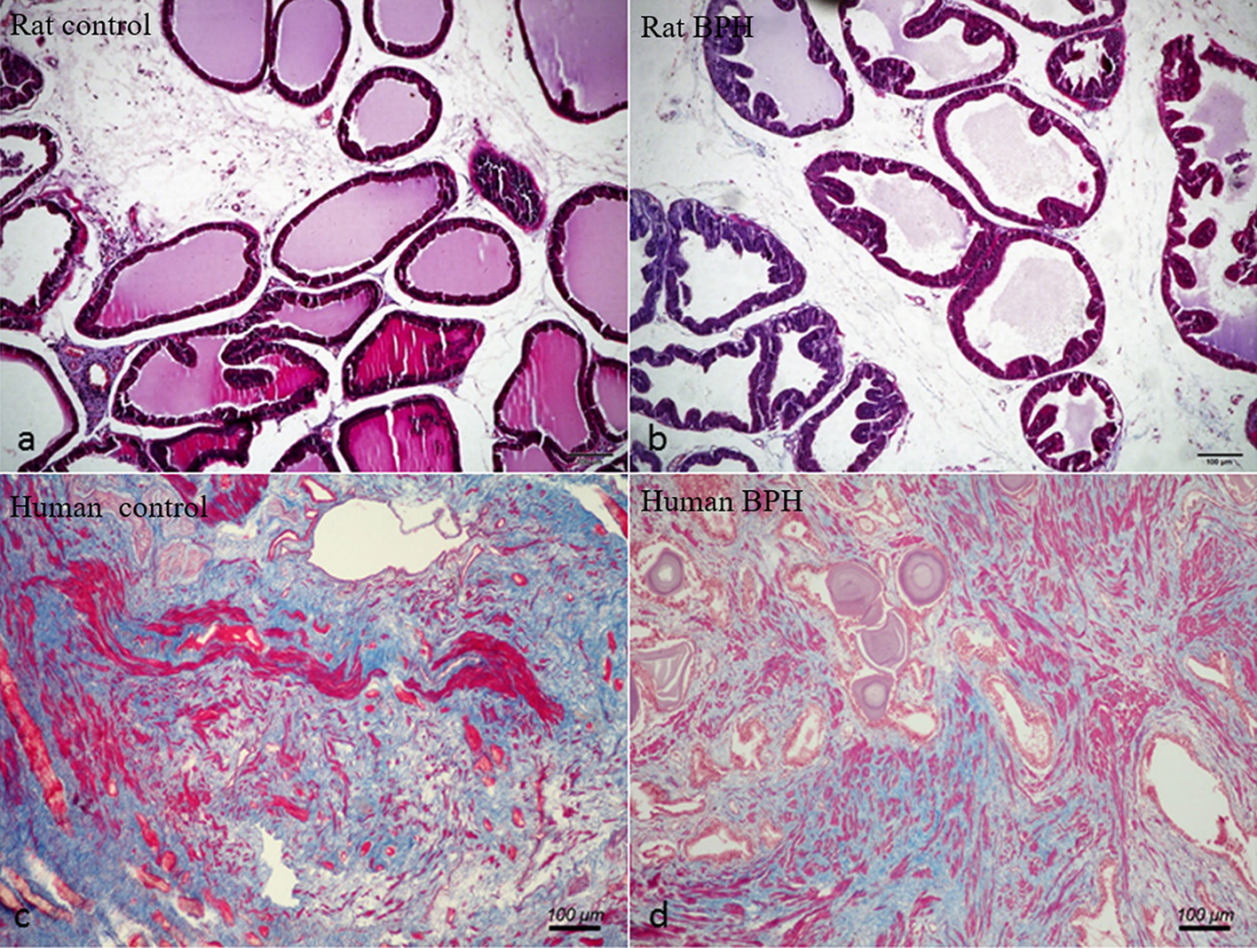
Masson's trichrome staining of prostate tissue. Prostatic SM cells were stained red, collagen fibers were stained blue and epithelial cells were stained orange. **(a)** Normal rat prostate. **(b)** BPH rat prostate. **(c)** Normal human prostate. **(d)** BPH human prostate (magnification × 100, *n* = 9 for each group).

**Figure 4:**
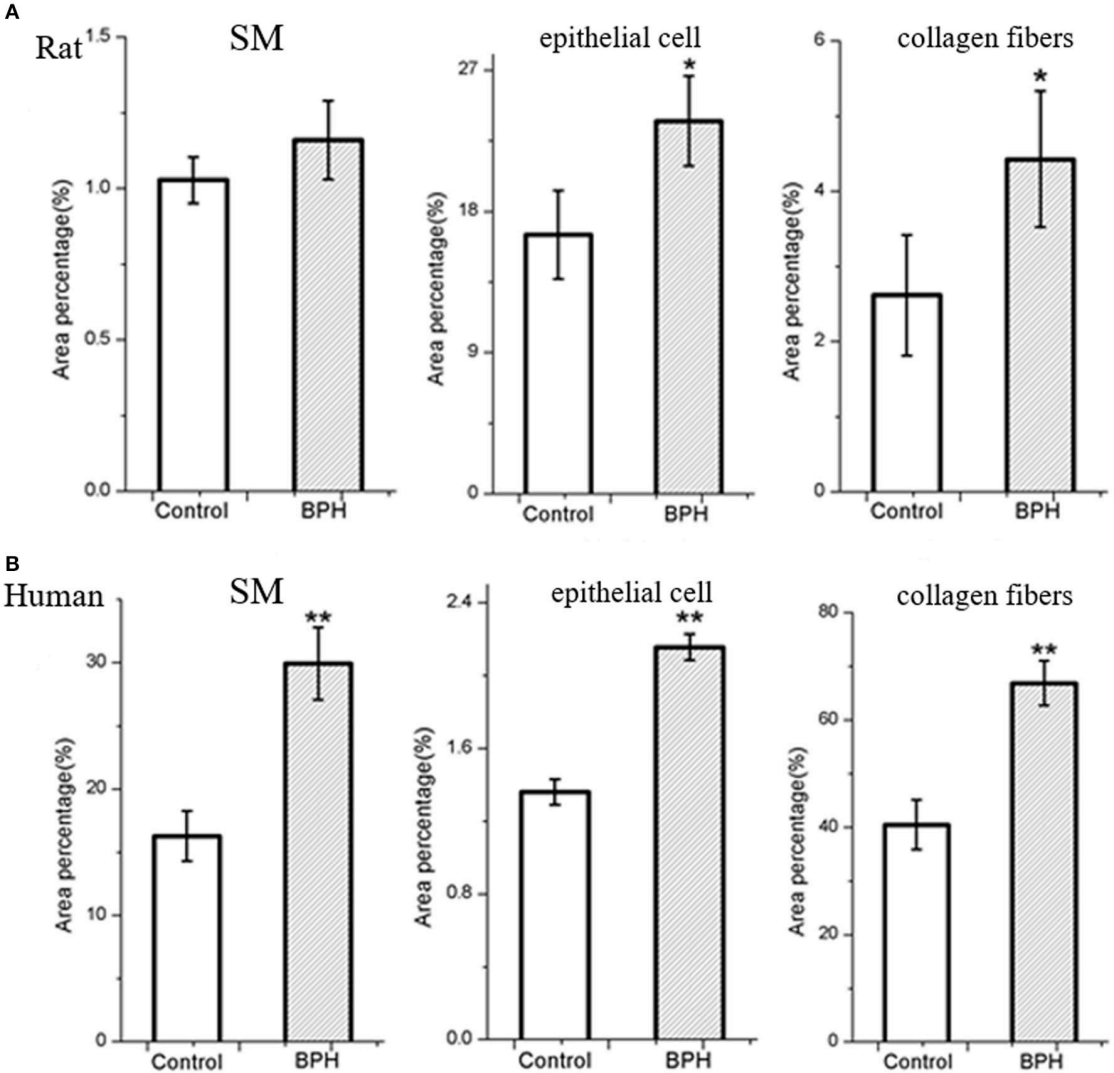
Area percentage of different components in normal and BPH group. The percentage of each component was quantified from three random 100 × fields of each tissue slice (*n* = 9 from each group). **(A)** Rat prostate. **(B)** Human prostate. Boxes, mean; bars, ± S.E.M; **P < 0.01 vs. control; *P < 0.05 vs. control.

Different histopathology was observed between human and rat BPH by H-E staining. In rat prostate, the hyperplasia mainly occurred in the epithelium including the epithelial layer thickened and papillary fronds protruded into the glandular cavities, with the stroma thickened concurrently (Figures 2a,b). In human prostate, an obvious stroma hyperplasia was observed, meanwhile the epithelial layer papillary fronds protruded into the glandular cavities (Figures 2c,d). With Masson's trichrome staining, prostatic SM cells were stained red, collagen fibers were stained blue and epithelial cells were stained orange (Figure 3). Compared to control group, all the constituents of the prostate augmented significantly except rat prostatic SM (Figure 4), in which only an increase trend was observed without statistical significance.

**Figure 5:**
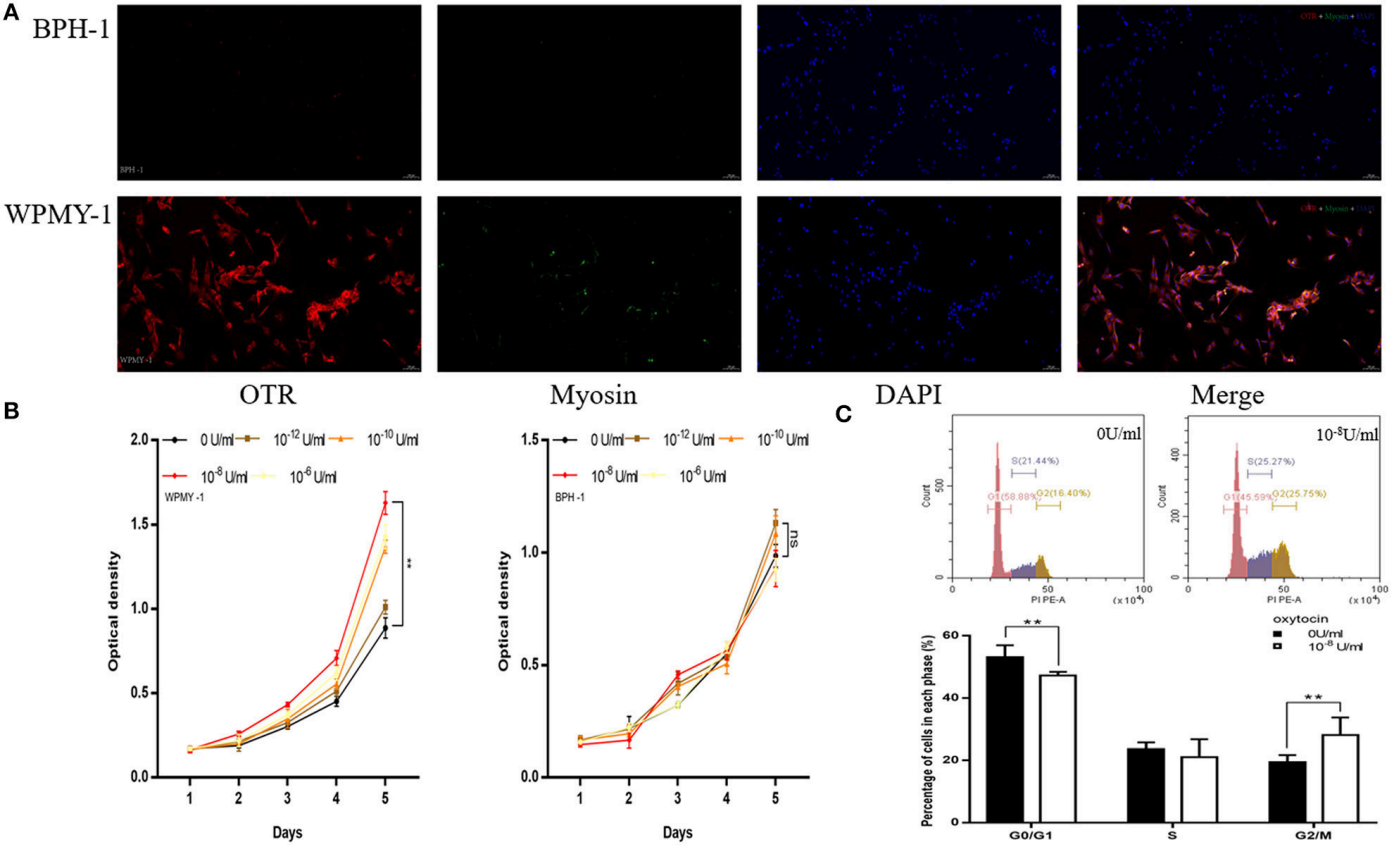
Immunofluorescence, cell proliferation and Flow Cytometry of human prostate cell lines. **(A)** Immunofluorescence of human prostate cell line. Cy3-immunofluorescence (red) indicates OTR expression. FITC-immunofluorescence (green) indicates MHC expression. DAPI (blue) indicates cell nuclear staining. Merged image indicates OTR, MHC and DAPI. **(B)** Cell proliferation of prostate cell treated with OT. X-axis is culture time and Y-axis is OD. **(C)** Flow Cytometry to analyze for cell cycle of WPMY-1 cells treated with OT. Upper: Flow Cytometry analyses for cell cycle in WPMY-1 cell treated with selected concentration of OT at 0 and 10^−8^ U/ml. Lower: Bar graph for the percentage of WPMY-1 cells in each cell phase. Cells cultured with 0 U/ml of OT are used as controls whereas cells cultured with other dosages of OT are the treatment groups. **P < 0.01 vs. control.

In the study of human cell immunofluorescence, myosin heavy chain (MHC) was stained green and was expressed in WPMY-1 cell (Figure 5A), which presented in human prostate stromal cells. OTR was stained red and also mainly expressed in WPMY-1 cells as well (Figure 5A). In contrast, MHC and OTR were not immunolocalized in the human epithelial cells. The CCK-8 assay revealed that OT can accelerate proliferation of stromal cell in a dose-depend manner from 10^−12^ to 10^−8^ U/ml (Figure 5B). At higher concentrations (10^−8^-10^−6^ U/ml), the proliferative effect was attenuated. Consistent with the OTR localization study, exogenous OT showed no effect on epithelial cell growth (Figure 5B). WPMY-1 cells were further treated with 10^−8^ U/ml OT for 48 h and cell cycle was determined with flow cytometry analysis. A significantly decreased cell proportion in the G_0_ and G_1_ cell division period was observed and a significantly increased cell proportion in G_2_ and M cell division period for WPMY-1 cells (Figure 5C).

**Figure 6:**
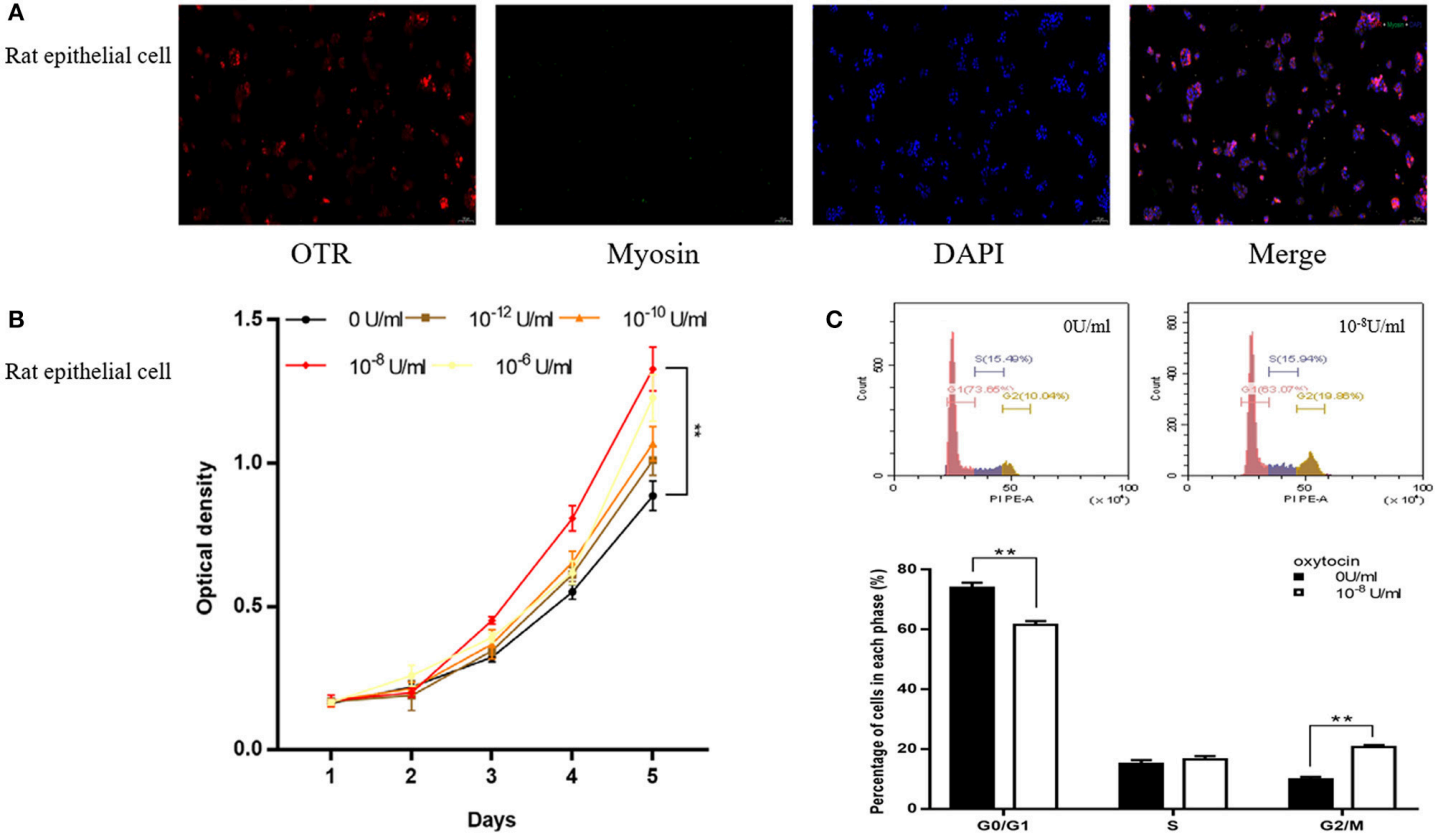
Immunofluorescence, cell proliferation and Flow Cytometry of rat primary epithelial cell. **(A)** Immunofluorescence of rat primary epithelial cell. Cy3-immunofluorescence (red) indicates OTR expression. FITC-immunofluorescence (green) indicates MHC expression. DAPI (blue) indicates cell nuclear staining. Merged image indicates of OTR and DAPI. **(B)** Cell proliferation of prostate epithelial cells treated with OT. X-axis is culture time and Y-axis is OD. **(C)** Flow Cytometry analyzing the cell cycle of rat epithelial cell treated with OT. Upper: Flow Cytometry analyses for cell cycle in rat epithelial cells treated with selected concentration of OT at 0 and 10^−8^ U/ml. Lower: Bar graph for the percentage of rat epithelial cells in each cell phase. Cells cultured with 0 U/ml of OT are used as controls whereas cells cultured with other dosages of OT are the treatment groups. **P < 0.01 vs. control.

In the study of rat cell immunofluorescence, myosin heavy chain (MHC) was not immunolocalized in rat epithelial cell (Figure 6A). OTR was stained red and expressed in rat primary epithelial cell (Figure 6A). The CCK-8 assay revealed that OT can accelerate proliferation of rat epithelial cell (Figure 6B). Rat primary epithelial cells were further treated with 10^−8^ U/ml OT for 48 h and cell cycle was determined with flow cytometry analysis. A significantly decreased cell proportion in G_0_ and G_1_ cell division period and a significantly increased cell proportion in G_2_ and M cell division period was observed for rat primary epithelial cells (Figure 6C).

**Figure 7:**
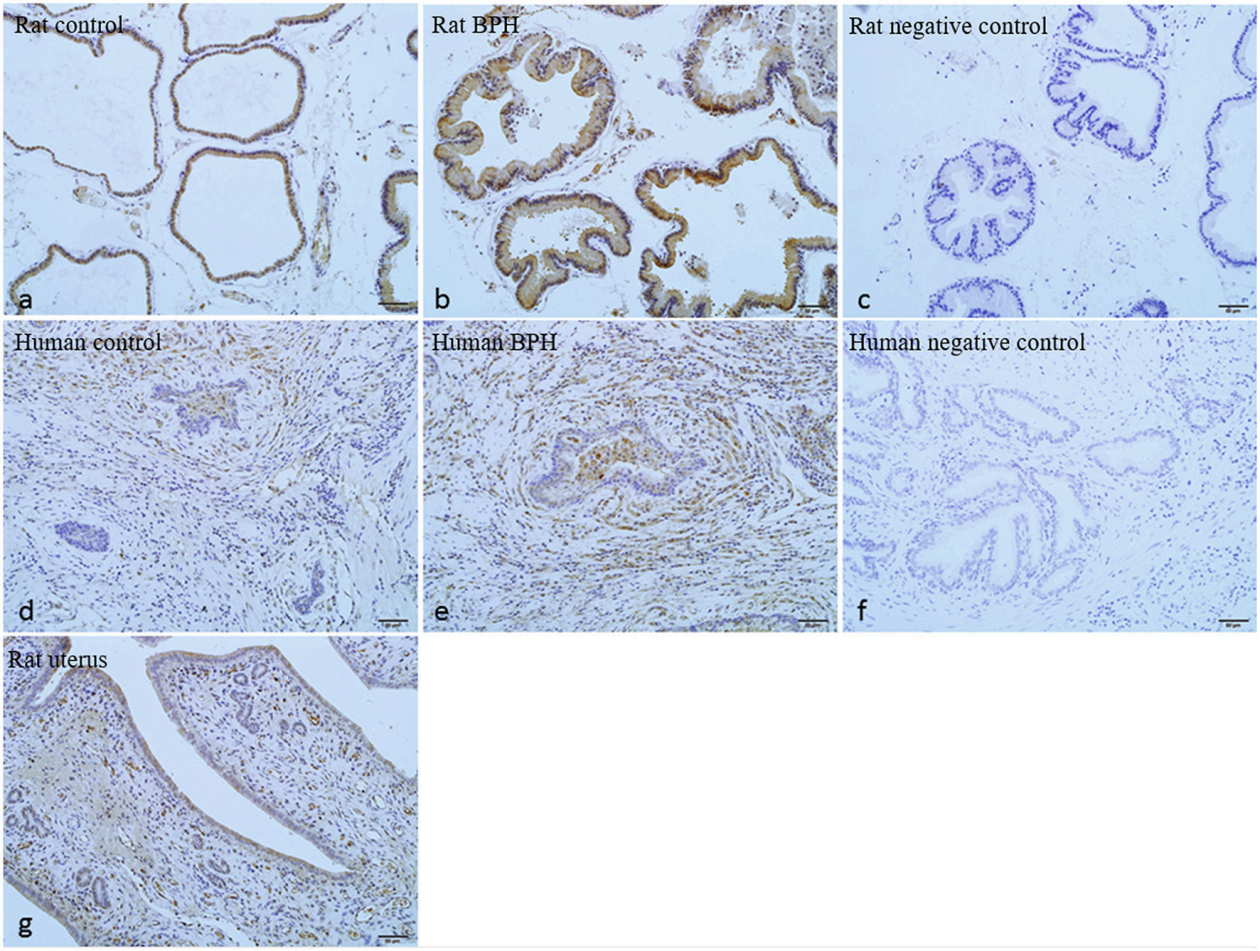
Immunohistochemistry of OTR. **(a)** Normal rat prostate. **(b)** BPH rat prostate. **(c)** Negative control of rat prostate. OTR mainly distributed in epithelium and partly distributed in stroma. **(d)** Normal human prostate. **(e)** BPH human prostate. **(f)** Negative control of human prostate. OTR mainly distributed in stroma. **(g)** Rat uterus tissue was used as positive control for OTR (magnification × 200).

**Figure 8:**
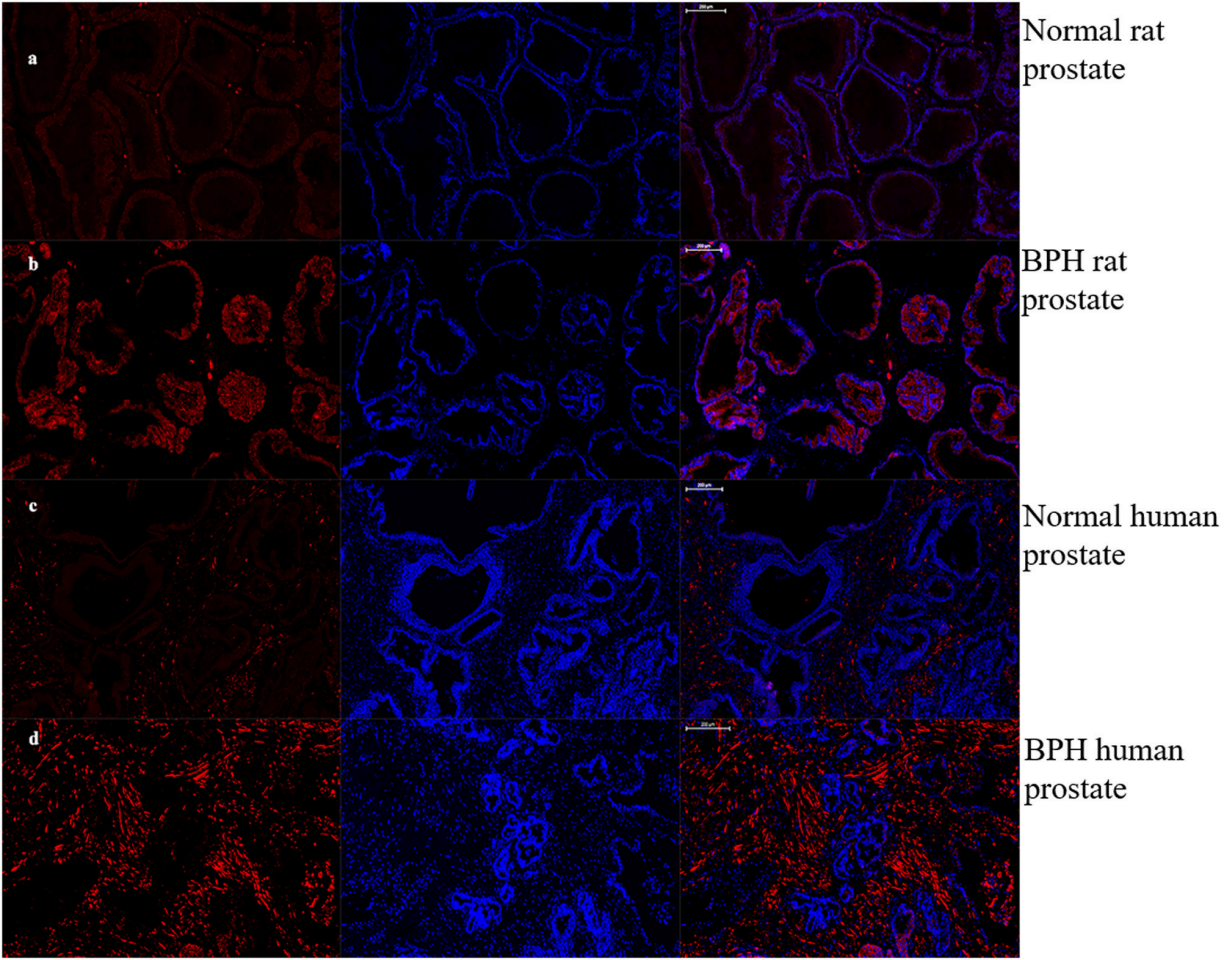
Immunofluorescence of OTR. Left: Cy3-immunofluorescence (red) indicates OTR was abundantly observed in prostate. Middle: DAPI (blue) indicates nuclear staining. Right: Merged image (magnification × 50). **(a)** Normal rat prostate. **(b)** BPH rat prostate. OTR mainly distributed in epithelium. **(c)** Normal human prostate. **(d)** BPH human prostate. OTR mainly distributed in stroma.

As shown in Figures 7, 8, OTR is localized in both human and rat prostate. In rat prostate, OTR was mainly present in the acinar epithelial cells and partly present in the stromal cells (Figures 7a,b). In contrast, OTR was predominantly present in the stromal cells for human prostate (Figures 7d,e). Negative controls omitting the primary antibody failed to stain (Figures 7c,f) and positive control using rat postpartum uterus tissue showed a strong immune positivity (Figure 7g). Immunofluorescence study showed OTR was similarly localized as immunohistochemistry (Figure 8).

**Figure 9:**
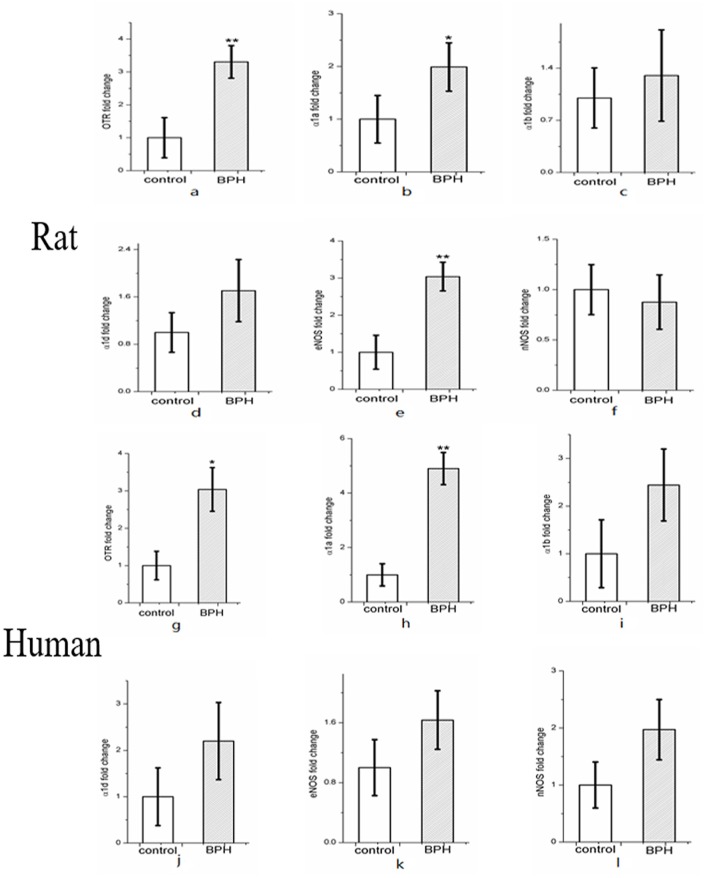
Expression of OTR **(a)**, α1-adrenoreceptor subtypes (α1aARs, α1bARs, α1dARs) **(b–d)** and nitric oxide synthase isoforms (eNOS and nNOS) mRNA **(e–f)** in rat (*n* = 15, 3 replicates per experiment) and human prostate **(g–l)** (*n* = 9, 3 replicates per experiment). Boxes, mean; bars, ± S.E.M; **P < 0.01 vs. control; *P < 0.05 vs. control.

Expression of OTR, α_1a_ARs, α_1b_ARs, α_1d_ARs, eNOS, and nNOS mRNA were determined using quantitative real time RT-PCR (Figures 9a–l). BPH upregulated OTR expression by approximately 3.3-fold in rat (Figure 9a) and 3.0-fold in human (Figure 9g) at the mRNA level (*P* < 0.01 in rat, *P* < 0.05 in human). Real time RT-PCR also showed that the expression of α_1a_ARs (*P* < 0.05) and eNOS (*P* < 0.01) was augmented significantly for rat BPH (Figures 9b,e) and α_1a_ARs (*P* < 0.01) was augmented significantly for human BPH tissue (Figure 9h). No change was observed for α_1b_ARs, α_1d_ARs, nNOS in rat BPH model and α_1b_ARs, α_1d_ARs, eNOS, nNOS in human BPH sample.

**Figure 10:**
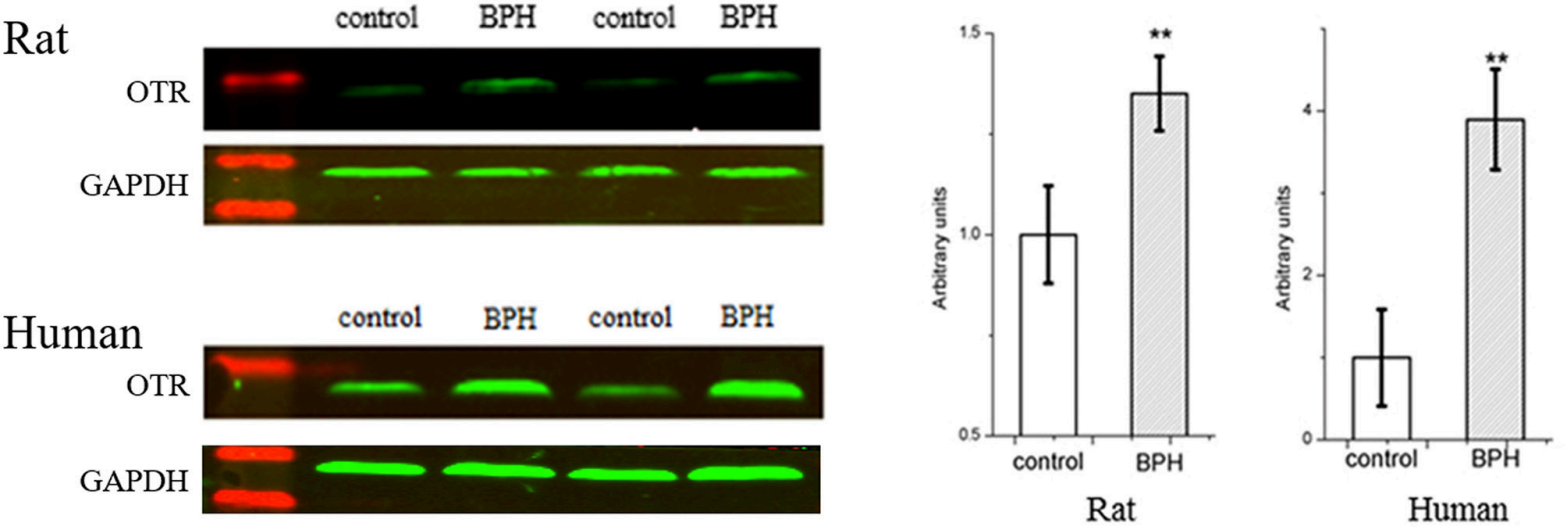
**Left**: Representative Western Blot bands of rat and human prostate. Two major specific bands of the expected sizes (37 (GAPDH) and 43 (OTR) kDa, green) are evident in all lanes, red bands indicate the protein markers. **Right**: Relative densitometric quantification of OTR in rat and human prostate. GAPDH expression was analyzed as a loading control, results are expressed as ratio of OTR to GAPDH. Boxes, mean; bars, ± S.E.M; **P < 0.01 vs. control.

Expression of OTR protein was further quantified with Western-blotting analysis (Figure 10). OTR protein bands was detected at a molecular weight of 43 kDa. A significant 1.4-fold and over 3.9-fold increase of OTR at translational level was observed for BPH rat (*P* < 0.01) and human sample (*P* < 0.01), respectively (Figure 10).

## Discussion

Our T-E induced BPH rat model was validated through hyperplasia of the prostate and seminal vesicle with both organ weights significantly increased. It was observed that the loss of body weight may be due to the exogenous T supplementation inducing more daily activity and an increased ratio of lean body mass/fat body mass for BPH rats (19). Previous studies demonstrated that a single T injection mainly led to an acinar epithelium hyperplasia of rat prostate (20, 21). In the current study, T-E injection induced stroma hyperplasia, although epithelia enlargement was still prominent. Moreover, the thickened stroma was mainly attributed to the increase of collagen, instead of SM cells. E was found to motivate some growth factors in prostate stromal cells both *in vivo* and *in vitro* (22–24). However, our Masson's trichrome staining showed the increase of SM in T-E treated rat prostate did not reach statistical significance. Therefore, it appears that the T-E induced BPH rat model still cannot mimic human BPH which is mainly a stromal hyperplasia.

The localization of OTR in prostate is varied in different studies or different species. OTR is mainly localized in the acinar epithelial cells in the prostate of dog, rat and possum (18). Furthermore, OTR is also localized to the basal layers of the secretory epithelium in the marmoset prostate (16). However, the immunoreactive receptor is observed mainly in stromal cells in human prostate (17). Our study of OTR localization is in agreement with previous studies in rat and human prostate. However, it contrasts with the findings by Whittington et al. that OTR immunoreactivity is present in epithelial cells in human hyperplastic prostate (4). This disparity in immunolocalization of OTR could be attributed to the different primary antibodies employed. In their study, Western-Blotting analysis detected a single band at 66 kDa whereas it was a single band at 43 kDa in our study. In the current study, immunoreactivity of OTR was also performed on human stromal cell line (WPMY-1) and epithelial cell line (BPH-1). Consistently, OTR was mainly expressed in stromal cells in human prostate.

For the first time, our data demonstrates an increased expression of OTR gene and protein in both rat and human hyperplastic prostate, with a more significant protein increase observed in human BPH than that in rat. We did not perform image quantification for OTR immunostaining since quantitative real time RT-PCR and Western-Blotting are more convincing measurements. However, the mechanism of OTR upregulation in BPH remains unclear. It has been found that both OT and OTR expressions are modulated by estrogen (25). Estrogen was shown to upregulate expression of OTR with dosage and time (26). Indeed, serum T may decrease but the ratio of E/T increased for aging males, especially in the prostate.

The effects of OT on cellular proliferation are tissue specific. In osteoblasts and human small cell carcinoma of lung, OT stimulates growth (13) whereas OT inhibits cellular proliferation in human breast cancer cells (27) and ovarian carcinoma cells (28). With regard to the rat and human prostate, the current study found OT stimulated the proliferation of rat epithelial cell and human stromal cell and had no effect on human epithelial cell, which was in agreement with immunostaining assay results that OTR was mainly expressed in rat epithelial cells and human stromal cells. Moreover, different concentrations of OT have different effects on human stromal cell proliferation. In our present study, the increasing dosage of OT from 10^−12^ to 10^−8^ U/ml induced proliferation with 10^−8^ achieving the strongest effect while the concentration of OT over 10^−8^ U/ml showed inhibitory effect on cell growth. Consistently, a previous study also observed that a lower concentration of OT can accelerate vaginal cell line Vk2E6E7 cell proliferation with a higher concentration inhibiting cell proliferation (29). Additionally, higher concentrations of OT may activate vasopressin V_1A_ receptor (30–32). Moreover, different dosage of OT may stimulate various OTR-mediated signaling pathways. In growth-inhibited cells, the effects of OT have been observed to be mediated by the non-conventional cAMP-protein kinase A pathway while in growth-stimulated cells, the effects were seen to be mediated by the “classical” increase in intra-cellular calcium and tyrosine phosphorylation (33). Furthermore, the lack of co-localization between OTR and caveolin-1 (CAV-1) might be a sign of desensitization of the OTR (25).

The cell cycle consists of four distinct phases: G_1_ phase, S phase (synthesis), G_2_ phase (collectively known as interphase), and M phase (mitosis) (34). Cells that have temporarily or reversibly stopped dividing are said to have entered a state of quiescence called G_0_ phase (34). G_0_ is a resting phase. G_1_ is from the end of the previous M phase until the beginning of DNA synthesis. G_2_ occurs after DNA replication and is a period of protein synthesis and rapid cell growth to prepare the cell for mitosis. Mitosis is the process by which a eukaryotic cell separates the chromosomes in its cell nucleus into two identical sets in two nuclei. Our FCM study demonstrated that more G_2_ and M phase of human prostate stromal cells were observed in response to treatment with OT, suggesting a pro-proliferation effect of OT.

Additionally, expressions of other molecular components involved in pathways associated with prostatic SM contraction and relaxation were determined. It was found that several of these genes were differentially modulated by BPH. In the rat model and human BPH samples, α_1a_ARs mRNA was significantly increased with no change for α_1b_ARs, α_1d_ARs. The rich expression of α_1a_ARs in the human prostate and excessive activity of α_1_-adrenergic SM contraction appears to be a common feature of symptomatic BPH (35). Moreover, previous quantification of α_1_ARs mRNA expression within human prostate has revealed that α_1a_ARs predominates, followed by α_1d_ARs and α_1b_ARs (36). In rat prostate, α_1_-adrenoceptor subtype is predominantly the α_1_A type (37). This predominance of α_1a_ARs is reportedly more marked in the hyperplastic prostate. The upregulation of eNOS in rat prostate may due to the morphological change in rat prostatic epithelium.

A limitation for our current study is that *in vitro* organ bath studies were not performed to explore the effect of OT on prostate contractility. Further study on OTR-mediated prostate tone will be intriguing. However, prostate SM contraction is thought to be predominantly induced by adrenergic receptors. OTR may possibly contribute mainly to cell proliferation, rather than weak contraction.

In conclusion, our novel data demonstrates that an increase of OTR gene and protein expression both in rat and human hyperplastic prostates. Furthermore, a lower dosage of OT can accelerate human stromal cell growth. Our study suggests that the upregulation of OTR could be one of the pathophysiological mechanisms of BPH and that the anti-OTR system could be a potential therapeutic target for BPH.

## Author contributions

ZL, HX, and KW contributed equally to this work. ZL and KW designed the experiment and wrote the first draft. PC and XW collected human prostate specimens. ZL, HX, and YZ finished the experiments. MD and XZ critically revised drafts of the manuscript. XZ provided important intellectual input and approved the final version for publication.

### Conflict of interest statement

The authors declare that the research was conducted in the absence of any commercial or financial relationships that could be construed as a potential conflict of interest. The reviewer RY and handling Editor declared their shared affiliation.
